# Long-lived antigen-induced IgM plasma cells demonstrate somatic mutations and contribute to long-term protection

**DOI:** 10.1038/ncomms11826

**Published:** 2016-06-07

**Authors:** Caitlin Bohannon, Ryan Powers, Lakshmipriyadarshini Satyabhama, Ang Cui, Christopher Tipton, Miri Michaeli, Ioanna Skountzou, Robert S. Mittler, Steven H. Kleinstein, Ramit Mehr, Frances Eun-Yun Lee, Ignacio Sanz, Joshy Jacob

**Affiliations:** 1 Emory Vaccine Center, Yerkes National Primate Center, Emory University, 954 Gatewood Road Atlanta, Georgia 30329, USA; 2 Interdepartmental Program in Computational Biology and Bioinformatics, Yale University, New Haven, Connecticut 74085, USA; 3 Lowance Center for Human Immunology and Division of Rheumatology, Department of Medicine, Emory University, Atlanta, Georgia 30329, USA; 4 Computational Immunology Lab, The Mina and Everard Goodman Faculty of Life Sciences, Bar-Ilan University, Ramat-Gan 5290002, Israel; 5 Department of Pathology, Yale University School of Medicine, Yale University, New Haven, Connecticut 74085, USA; 6 Department of Immunobiology, Yale University School of Medicine, Yale University, New Haven, Connecticut 74085, USA

## Abstract

Long-lived plasma cells are critical to humoral immunity as a lifelong source of protective antibodies. Antigen-activated B cells—with T-cell help—undergo affinity maturation within germinal centres and persist as long-lived IgG plasma cells in the bone marrow. Here we show that antigen-specific, induced IgM plasma cells also persist for a lifetime. Unlike long-lived IgG plasma cells, which develop in germinal centres and then home to the bone marrow, IgM plasma cells are primarily retained within the spleen and can develop even in the absence of germinal centres. Interestingly, their expressed IgV loci exhibit somatic mutations introduced by the activation-induced cytidine deaminase (AID). However, these IgM plasma cells are probably not antigen-selected, as replacement mutations are spread through the variable segment and not enriched within the CDRs. Finally, antibodies from long-lived IgM plasma cells provide protective host immunity against a lethal virus challenge.

## Abstract (web summary)

Long-lived IgG plasma cells develop in germinal centres and then home to the bone marrow and persist for a lifetime. Here the authors identify long-lived IgM plasma cells in the murine spleen, which carry IgH mutations but can develop independently of germinal centres, and confer protective antiviral immunity.

## Graphical abstract

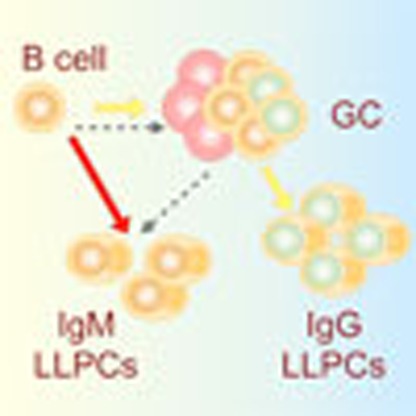

Immune memory can last a lifetime, in no small part due to antigen-specific, long-lived plasma cells (LLPCs) that continuously secrete antibodies and provide long-term protection^1^,^2^,^3^,^4^,^5^. These plasma cells (PCs) develop from antigen-specific B cells through one of two pathways, resulting in either short-lived PCs or LLPCs. Following activation, antigen-specific B cells are able to progress immediately into short-lived PCs without T-cell help. These short-lived cells exist only transiently in the spleen and lymph nodes, and do not undergo affinity maturation^3^,^5^. In contrast, LLPCs are of higher affinity than their short-lived counterparts and can survive for many years. These LLPCs are generated within the germinal centres (GCs), which are sites of intense proliferation and affinity maturation of antigen-specific B cells. These GC B cells differentiate into either memory B cells, which remain in the spleen, or IgG LLPCs that home to the bone marrow and persist for a lifetime^1^,^2^,^3^. IgG LLPCs, rather than memory B cells, are the key source of long-term IgG secretion^1^,^6^.

The development of both memory B cells and IgG LLPCs is significantly impaired in the absence of GC formation^7^,^8^. The interaction between CD40 on B cells and CD40L on T-helper cells is essential to GC function. It has been shown that blockade with αCD40 antibodies impairs memory B cell formation^7^, and that αCD40L antibody treatment blocks the development of IgG LLPCs^8^, and both studies show significantly reduced long-term IgG titres.

Following activation, B cells clonally expand and undergo the process of class-switching and somatic hypermutation (SHM)^9^,^10^. During class-switching, B cells switch from surface IgM expression to IgG, IgA and IgE isotypes. These cells also undergo SHM and antigen selection^4^,^9^. SHM is the process by which point mutations accumulate in the immunoglobulin (Ig) variable loci. This is initiated by activation-induced cytidine deaminase (AID), which generates a pool of antigen-specific B cell clones with varying affinities and, out of these, high-affinity clones are selected by antigen. This process typically selects for replacement mutations (mutations resulting in an amino acid change) at higher frequency within the antigen-binding, complementary determining regions (CDRs) than in framework regions, as is commonly seen in both memory B cells and IgG LLPCs^4^,^11^.

Here we report that a distinct population of antigen-specific, induced IgM LLPCs persists in the murine spleen, in response to either vaccination or infection, and secretes high titres of antigen-specific IgM throughout the lifetime. These cells, unlike IgG LLPCs, are able to develop and somatically mutate even in the absence of GCs. These mutations were found in AID-induced hotspots^4^, but unlike IgG PCs did not show evidence of antigen-selection. In addition, IgM LLPCs were found to protect against viral challenge *in vivo*.

## Results

### IgM LLPCs persist in the spleen

**Figure 1:**
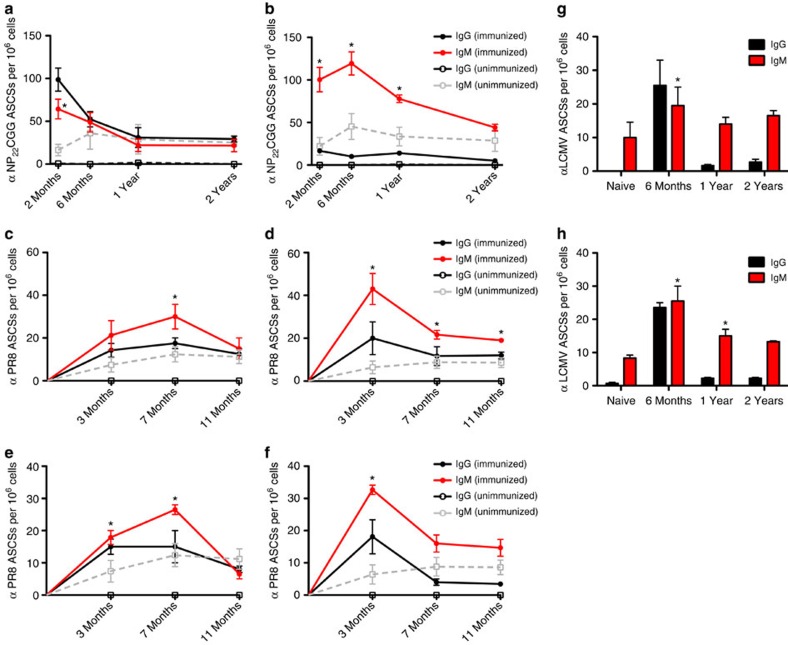
Expanded populations of IgM PCs persist in the spleen in response to diverse immunogens and pathogens. Cohorts of mice were immunized or infected with NP_22_CGG, influenza or LCMV virus, and IgG (black circles) and IgM (red circles) ASCs from immunized and unimmunized (black squares, IgG; grey squares, IgM) were measured via ELISPOT over time. (**a**,**b**) NP_22_CGG-specific IgG and IgM PCs at 2 months, 6 months, 1 year and 2 years post immunization (*n*=6) or in naive controls (*n*=3) were quantified in the bone marrow and spleen, respectively. (**c**,**d**) A/PR/8/34 influenza virus-specific PCs at 3, 7 and 11 months post infection with 0.01xLD_50_ of virus (*n*=4) or in naive controls (*n*=3) within the bone marrow and spleen, respectively. (**e**,**f**) A/PR/8/34-specific PCs at 3, 7 and 11 months post immunization with 1,400 HA of virus (*n*=4) or in naive controls (*n*=3) within the bone marrow and spleen, respectively. (**g**,**h**) LCMV-specific IgG and IgM PCs in the bone marrow and spleen, respectively, at 6 months, 1 year and 2 years post infection with 2 × 10^5^ p.f.u. of LCMV Armstrong virus compared with a 6-month naive control (*n*=3). The mean (±s.e.m.) is shown for each timepoint and an asterisk (*) indicates time points where IgM ASCs are significantly higher (*P*<0.05, Student's *t*-test) than in naive animals.

We initially sought to compare IgG PC longevity in the bone marrow compartment over a time course of up to 2 years following protein immunization. As a control, we also quantified IgM PCs in the spleen with the expectation that these would be short lived. Age-matched naive cohorts served as control for natural IgM cells. Natural IgM cells produce natural IgM antibodies, which are constantly present in the serum and provide an early, polyreactive response against pathogens^12^. To assess longevity, we first immunized mice with hydroxy-3-nitrophenylacetyl chicken-γ-globulin (NP_22_CGG) in alum and quantified antigen-specific antibody-secreting cells (ASCs) by enzyme-linked immunospot (ELISPOT) in both the bone marrow and spleen. We found comparable numbers of antigen-specific ASCs in both the bone marrow (Fig. 1a) and spleen (Fig. 1b) persisting at 2 months, 6 months, 1 year and even 2 years post immunization. Interestingly, when we compared IgM ASCs in the immunized cohorts to the naive controls, we found a significant number of long-lived, antigen-specific IgM ASCs in the spleen. These long-lived IgM ASCs were found in significantly higher numbers than both IgG and natural IgM cells seen in naive controls and persisted almost 2 years following immunization (*P*<0.05, *t*-test for all time points up to 1 year). As with IgG LLPCs, we observed an initial spike in IgM ASCs in immunized mice, followed by a slow decay over the lifetime of the animal. By contrast, natural IgM ASCs accumulated in mice over time, up to around 6 months, and then remained relatively constant for the rest of their lifespan. To confirm that this observation on long-lived, induced IgM PCs was not merely an artefact of the hapten-carrier system, we also infected (Fig. 1c,d) or immunized mice (Fig. 1e,f) with live or betapropiolactone (BPL)-inactivated A/Puerto Rico/8/1934 influenza virus (PR8), respectively. Again we found similarly long-lived IgM responses in the spleen, significantly above naive controls, in particular in the infected cohorts. We additionally extended these studies to infection with the Armstrong strain of lymphocytic choriomeningitis virus (LCMV) (Fig. 1g,h) and again observed significantly higher numbers of antigen-specific, IgM LLPCs in the spleen compared with naive controls.

### IgM PCs persist in the spleen post-adoptive transfer

**Figure 2:**
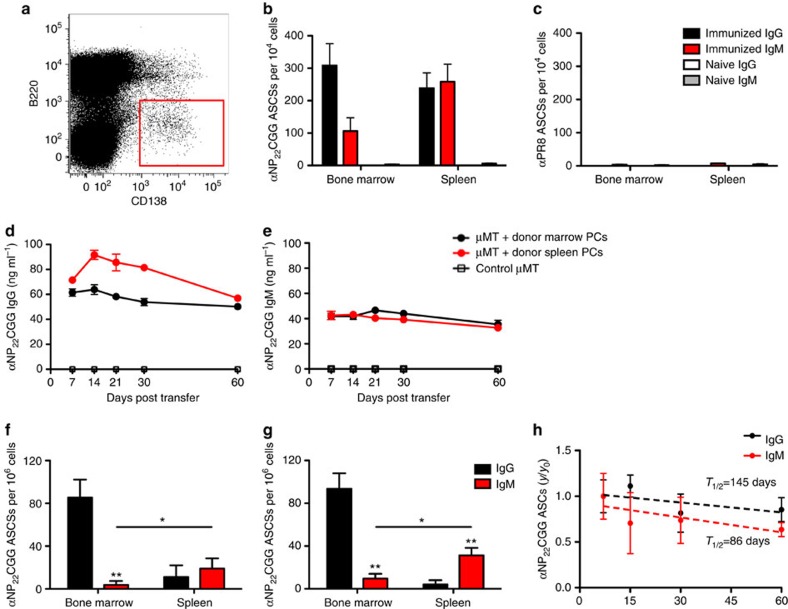
Antigen-specific, IgM LLPCs persist post-adoptive transfer and preferentially localize in the spleen. (**a**) Representative CD138^+^B220^*−*^ PC gating strategy for FACS. (**b**) NP_22_CGG-specific IgG (filled black square) and IgM (filled red square) ASCs in 10^4^ sorted PCs, 2 months post-NP_22_CGG immunization (*n*=3) or in age-matched controls (open black square, *n*=3). (**c**) Nonspecific (A/PR/8/34 binding) ASCs in 10^4^ sorted PCs, 2 months post-NP_22_CGG immunization (*n*=3) or in age-matched controls (*n*=3). (**d**) Antigen-specific serum IgG titres found in recipient μMT mice that have been adoptively transferred with bone marrow PCs (black circles) or splenic PCs (red circles) as measured by ELISA at days 7 through 60 (*n*=5). (**e**) Antigen-specific serum IgM titres found in recipient μMT mice that have been adoptively transferred with bone marrow PCs (black circles) or splenic PCs (red circles) as measured by ELISA at days 7 through 60 (*n*=5). (**f**) Localization of donor, NP_22_CGG-specific IgG (filled black square) and IgM (filled red square) ASCs, adoptively in bone marrow PC recipient mice. (**g**) Localization of donor, NP_22_CGG-specific IgG (filled black square) and IgM (filled red square) ASCs, adoptively in splenic PC recipient mice. The mean (±s.e.m.) of five immunized mice or three naive controls is shown, with ** indicating Student's *t*-test *P*<0.005. (**h**) The half-life of transferred cells was determined by fitting the time-course data of either IgG or IgM PCs in splenic recipient mice (*n*=4) as a function of their ratio (*y*/*y*_initial_) with its linear regression.

To determine whether IgM PCs are inherently long lived, we sorted 10^4^ splenic or bone marrow CD138^+^B220^*−*^ PCs and adoptively transferred cells into B-cell-deficient μMT mice or lymphocyte-deficient *Rag*^*−/−*^ mice. To demonstrate that IgM PCs are antigen specific, and when transferred did not contain natural IgM, we assessed the specificity of sorted PCs via ELISPOT (Fig. 2a). Only PCs from immunized mice bound NP_22_CGG (Fig. 2b) and neither immunized nor naive PC populations reacted with irrelevant antigen—PR8 influenza virus (Fig. 2c). We hypothesized that if IgM PCs were inherently short lived, we would observe a greater decay of IgM antibody titres in recipient μMT mice. However, we found comparable NP_22_CGG-specific IgG and IgM antibody titres post-adoptive transfer as measured by enzyme-linked immunosorbent assay (ELISA) (Fig. 2d,e), indicating that IgM PCs are not inherently short lived. We observed similar long-lasting titres of IgM antibodies post transfer into *Rag−/−* recipient mice (Supplementary Fig. 1). To confirm IgM PC survival, we killed the recipients after 2 months and assayed for the presence of IgG and IgM PCs in the spleen and bone marrow by ELISPOT. Our results demonstrate that antigen-specific IgM PCs survive 2 months post transfer. Interestingly, our results indicate a marked preference for IgG LLPCs to localize to the bone marrow and IgM LLPCs to localize to the spleen (Fig. 2f,g).

To further characterize the longevity of splenic and IgM PCs, we determined their half-life via time-course ELISPOT (Fig. 2h). By transferring CD138^+^B220^*−*^ PCs from donor mice at 2 months post immunization, we have not only excluded short-lived PCs but also natural IgM cells (Fig. 2b,c) from our analysis. We then followed the decay of splenic IgG and IgM cells over the course of two months and determined the half-life of each population from its linear regression. We were unable to determine using 5-bromodeoxyuridine labelling whether these cells were still undergoing proliferation, owing to the paucity of these PCs following transfer. However, we did estimate the half-life of IgG and IgM PCs transferred from the spleen to be *t*_1/2_=145 days and *t*_1/2_=86 days, respectively. The half-life of these IgM PCs is relatively long compared with their short-lived counterparts and the rate of decay is not significantly different than that of IgG PCs (*P*=0.82 by *t*-test).

### IgM LLPCs develop in the absence of GCs

**Figure 3:**
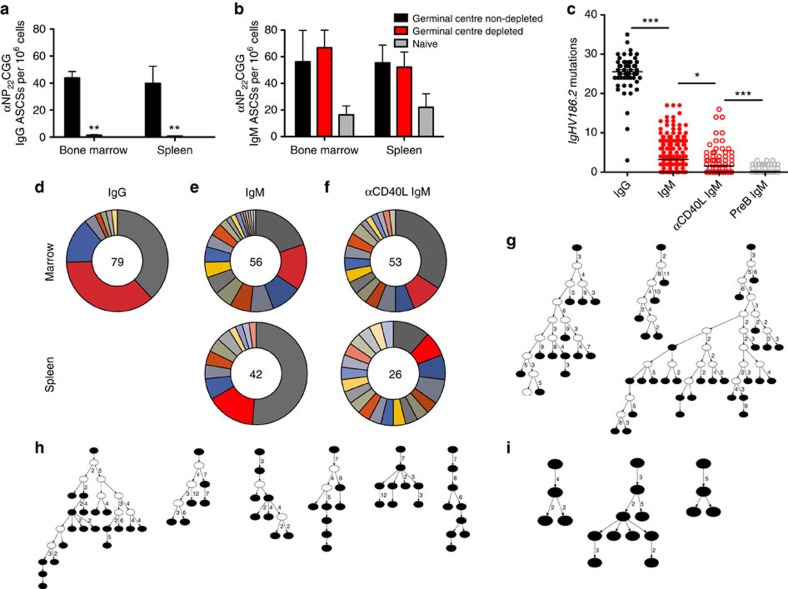
IgM PCs show evidence of SHM even when GCs are ablated. (**a**,**b**) NPCGG-specific IgG (filled black square) and IgM (filled red square) ASCs in mouse cohorts treated with either control IgG (*n*=5), αCD40L antibody to deplete GCs (*n*=7), or in age-matched naive controls (filled red square, *n*=3), as measured via ELISPOT at day 45 post immunization. The mean (±s.e.m.) is shown with ** indicating *P*<0.005. (**c**) IgG (black circles) and IgM *IgHV186.2* sequence mutations in cohorts of C57/BL/6 mice at 1 and 3 months post-NP_22_CGG immunization (pooled), with (red open circles) or without (red circles) αCD40L treatment, compared with total heavy chain sequences from pre-B-cell unmutated controls (open grey circles) isolated from immunized C57/BL/6 cohorts (*n*=4). Spleen and bone marrow PCs are pooled; Student's *t*-test *P<0.05 and ***P<0.001. Refer to Supplementary Table 1 for sequence numbers. (**d**) Representative clonality of C57/BL/6 bone marrow IgG PCs post immunization—each section represents a shared CDR3 junction as a percentage of total sequences as listed in the centre. Representative clonality of C57/BL/6 IgM PCs in the spleen and bone marrow, treated with control Ig (**e**) or with αCD40L (**f**). Ig lineage trees for IgG (**g**) and IgM PCs, treated with with control Ig (**h**) or with αCD40L (**i**). Filled nodes represent germline or sample sequences; empty nodes indicate inferred precursors. Each line denotes either a single mutation between parent and daughter if no number is given.

Bone marrow-resident IgG LLPCs are dependent on GCs for their development. Disruption of GCs by blocking T-cell help via αCD40L antibody treatment^8^, depleting complement by cobra venom factor (CVF)^13^, or the use of gene knockout animals^14^ completely ablates the IgG LLPC pool. To determine whether IgM LLPCs are GC dependent, we administered αCD40L antibodies at days 6, 8 and 10 following immunization, to block the formation of the GC. As expected^8^, IgG LLPCs were ablated following GC disruption (*P*<0.005 by *t*-test) (Fig. 3a). Surprisingly, depletion of GCs did not impair the formation of IgM LLPCs (Fig. 3b). We also used a second approach to disrupt GCs by treatment with CVF and observed that the development of IgM LLPCs still occurs while IgG LLPCs are significantly impaired (Supplementary Fig. 2). Taken together, this suggests that induced IgM LLPCs, unlike their IgG counterparts, can develop even in the absence of GCs.

### IgM LLPCs are somatically mutated

SHM is a hallmark of IgG LLPCs in the bone marrow^4^,^9^,^15^. Thus, we wanted to determine whether IgM LLPCs exhibited mutations in their Ig loci, analogous to IgG LLPCs. However, PCs lack surface Ig and hence they cannot be sorted based on antigen specificity. To overcome this limitation, we immunized mice with NP_22_CGG, which induces a well-documented response in which the *IgHV186.2* gene segment in Igh^b^ mice encodes NP specificity^16^,^17^. Briefly, at 1 or 3 months post immunization, and with and without GC depletion via αCD40L treatment in C57/BL/6 cohorts, we have amplified and sequenced those cells expressing the canonical *IgHV186.2* variable segment. We then analysed these sequences using The International Immunogenetics Information System's (IMGT) HighV-QUEST^18^, to identify the germline heavy chain *V(D)J* gene segments and map mutations by comparison with a database of known sequences. Supplementary Table 1 shows the numbers of full-length sequences and unique clones found in each animal with a >90% homology to *IgHV186.2*. As a control, we also sequenced the heavy chains of pre-B cells isolated from the bone marrow of these same mice. A small number of *IgHV186.2* sequences were found in the pre-B population, all identical to the published germline sequence. Although there were some rare instances of diverging base pairs seen in the total pre-B population, these did not result in amino acid changes. *IgHV186.2* IgG from bone marrow-resident LLPCs showed an abundance of mutations, with an average of 25.5 mutations per sequence. IgM LLPCs showed significantly fewer mutations, with an average of 3.3 mutations per sequence, and a third of sequences remained unmutated. In addition, with GC depletion there was a small but significant decrease in the number of mutations observed in IgM LLPCs—on average 2.1—although they were still significantly mutated when compared with pre-B controls (average 0.4) (*P*<0.001 by *t*-test) (Fig. 3c). As a control, we also sorted out CD138^+^B220^*−*^ PCs from unimmunized mice and attempted to amplify *IgHV186.2V* gene segments, but these rearrangements could not be found. Combined with the absence of NP-specific ASCs in the sorted CD138^+^B220^*−*^ PCs from unimmunized mice (Fig. 2b), this data further illustrate that IgM LLPCs are induced rather than natural IgM cells.

During B-cell development and assembly of the B-cell receptor from Ig gene segments, imprecise joining of the V, D and J segments creates a unique junction at the CDR3 region^19^. These unique CDR3s can be used to identify individual clones. Figure 3d,e illustrates the clonality of representative PC populations detailed in Supplementary Table 1. Each clone is identified by a unique CDR3 junction and may have multiple members based on diversification by SHM. In a representative mouse, 79 individual IgG sequences from bone marrow PCs contained 7 clonal populations, with 3 clones accounting for 89% of the sequences (38%, 36% and 15%, respectively), showing significant levels of clonal expansion (Fig. 3d). We see similar evidence of clonal expansion in IgM PC sequences, with and without αCD40L treatment, although the effect is less pronounced in some samples (Fig. 3e,f). Fifty-six IgM heavy chains from the bone marrow of a representative mouse can be grouped into 24 unique clones, with the 3 largest clones comprising 45% of all sequences (20, 15 and 10%). Forty-two IgM heavy chains from the spleen include 11 unique clones, with the 3 largest comprising 74% of all sequences (51, 16 and 7%). Fifty-three αCD40L-treated bone marrow IgM sequences contained 17 clones, with the 3 largest comprising 50% of total sequences (34, 10 and 6%), whereas 26 αCD40L spleen IgM sequences contained 21 unique clones with the 3 largest comprising 17% of sequences (12, 8 and 8%). Within individual clones, the diversification via SHM and the establishment of parent–daughter clones can be visualized by constructing lineage trees. We have constructed these trees for both IgG and IgM PC clones, with and without GC depletion, using IgTree software^20^ (Fig. 3g–i). These lineage trees demonstrate that the mutational diversity generated within a common B-cell clone persists in the periphery, for both IgG and IgM LLPC subsets. This indicates that the mutants generated at the activated B-cell stage were not lost but many were selected for and can be found in the PC compartments. These trees also further illustrate the clonal expansion that has taken place in both IgG and IgM LLPC populations, although there are fewer mutations in IgM clones—and fewer still without GC help—mirroring the pattern we see in all *IgHV186.2*-encoded LLPCs.

### Mutations occur in motifs preferentially targeted by AID

**Figure 4:**
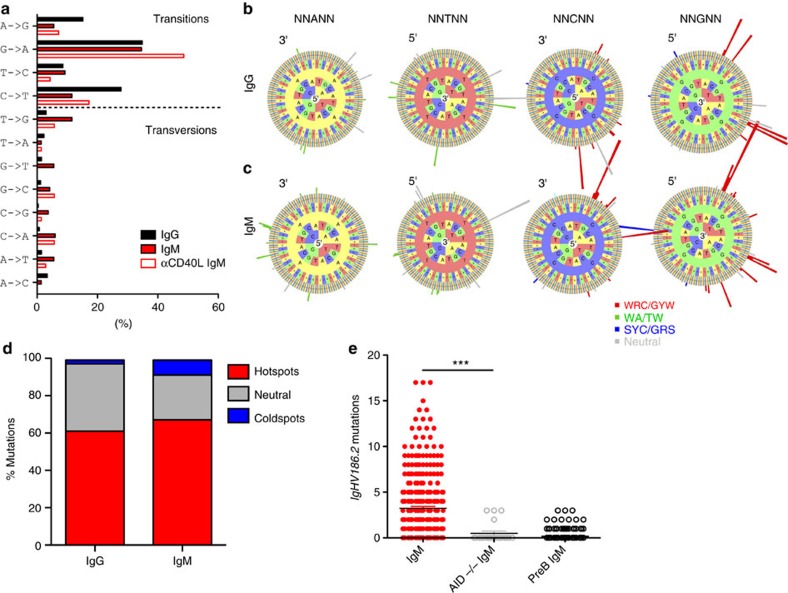
IgM heavy chain mutations are AID-induced and AID-dependent. (**a**) Frequency of transition and transversion mutations for IgG (filled black square) and IgM PCs, with (open black square) or without (filled red square) αCD40L GC depletion. (**b**,**c**) Individual 5-mer mutabilities estimated from IgG and IgM sequences, respectively. Bars represent mutabilities of the central base as a function of the surrounding bases, read 5′–3′. Green (WA/TW) and red (WRC/GYW) bars indicate hotspot motifs, grey indicates the neutral sites and blue indicates the coldspots (SYC/GRS). Analysis was performed on total *IgHV186.2* sequences for each population. (**d**) Percentage of total mutations found hotspot motifs (WRC/GYW or WA/TW) in red, neutral sites in grey, or coldspot motifs (SYC/GRS) in blue for IgG and IgM sequences. (**e**) IgM *IgHV186.2* sequence mutations in cohorts of C57/BL/6 (filled red circles, *n*=4) or AID^*−*/*−*^ (open grey circles, *n*=3) mice post-NP_22_CGG immunization, compared with total heavy chain sequences from pre-B-cell unmutated controls (open grey circles) isolated from immunized C57/BL/6 cohorts. Spleen and bone marrow PCs are pooled; and Student's *t*-test *P<0.05 and ***P<0.001. Refer to Supplementary Table 1 for sequence numbers.

We next sought to determine whether mutations in IgM sequences were introduced by the SHM mechanism. SHM is initiated by AID, which mutates DNA by deaminating the cytidine base, creating a uracil. Uracil-DNA glycosylase and apurinic endonuclease are then involved in excising uracil out and DNA repair mechanisms fill the gap^21^,^22^. This process preferentially creates transition mutations (C<–>T and G<–>A) over transversions (all other changes)^4^. Here we compared the frequency of transitions versus transversions in IgG and IgM PCs, with and without GC depletion. Transitions were much more common for all populations compared with transversions in *IgHV186.2* sequences—characteristic of AID-induced mutation^4^,^15^,^23^ (Fig. 4a). To further testify the involvement of the AID-induced SHM mechanism, we analysed whether mutations in IgG and IgM PCs occurred in SHM ‘hotspot' motifs, where mutations are most likely to occur. The known hotspots are WRC/GYW and WA/TW, where the underlined nucleotide is mutated, W=weak (A,T), R=purine (A,G) and Y=pyrimidine (T,C). In contrast, SYC/GRS is considered a coldspot, where S=strong (G,C) and other motifs are considered ‘neutral'^24^,^25^. The data are depicted as ‘hedgehog' plots that show individual IgG (Fig. 4b) and IgM (Fig. 4c) 5-mers, with sequences read 5′–3′, and the radiating bars indicating mutability at that motif. Mutations occur at the central base and are dependent on surrounding bases, with classical hotspots indicated by red or green bars, coldspots by blue and neutral sites by grey. Figure 4c shows the cumulative hotspot and coldspot mutabilities as a percentage of total mutations. Both IgG and IgM PC sequences predominantly exhibited mutations in hotspots (>60%) and, to a lesser degree, in coldspots (<7%), with no significant differences seen between IgG and IgM PCs. Taken together, these data indicate that the pattern of mutation in IgM PC is similar to that of IgG.

### Mutations in IgM LLPCs are AID dependent

**Figure 5:**
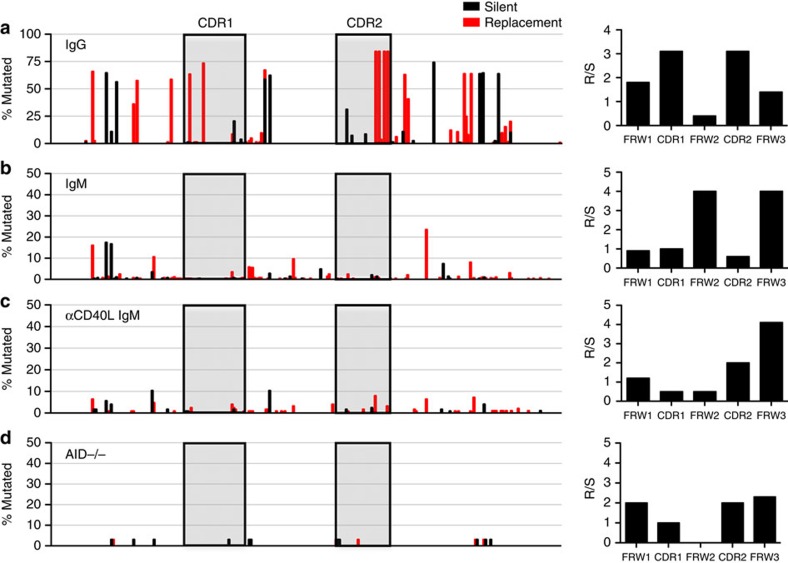
IgM heavy chain mutations are not enriched in CDR regions and show no evidence of antigen selection. Mutational frequency or replacement (in red) and silent mutations are plotted for *IgHV186.2* regions of IgG (**a**) and IgM (**b**) PCs, as well IgM PCs with αCD40L blockade (**c**) and AID^*−*/*−*^ IgM PCs (**d**), with replacement to silent amino acid changes (R/S ratios) given to the right.

Having demonstrated that mutations in IgM occur predominately in AID-targeted hotspots, we wanted to confirm that AID was the primary mechanism of these mutations. It has been previously shown that in AID-knockout mice, class-switching does not occur and IgG LLPCs do not develop^26^. We immunized cohorts of AID−/− mice with NP_22_CGG and observed that the formation of induced IgM LLPCs was not impaired in these mice. We then sequenced sorted IgM LLPCs from AID−/− cohorts and wild-type C57/BL/6 mice, alongside pre-B controls. We found that mutations did not occur in AID^*−*/*−*^ cohorts, equivalent to what we observed in pre-B cell controls (Figs 4e and 5d). This data demonstrates that mutations found in IgM LLPCs are purely AID induced.

### Mutations in IgM LLPCs are probably not antigen selected

Having demonstrated that IgM LLPCs accumulate somatic mutations, we next analysed the position of these mutations, to compare the extent of antigen selection in Ig heavy chains of IgG and IgM LLPCs. Antigen selection typically enriches for replacement mutations within the CDRs, which bind antigen, whereas mutations that disrupt the framework structure are not tolerated^4^,^11^. When we mapped out the replacement and silent mutations that occur in sample bone marrow IgG LLPCs, we observe a high frequency of replacement mutations within CDR1 and CDR2, as expected (Fig. 5a). In addition, we observed a clear pattern of increased replacement to silent mutation (R/S) ratios in CDRs regions (R/S >3) compared with those in framework regions (R/S <2). No significant mutations are seen in AID−/− mice (Fig. 5d). However, although a greater number of mutations are seen in IgM LLPCs of wild-type mice, we did not observe a high frequency of mutations, especially replacement mutations, in the CDRs of IgM. Furthermore, the R/S ratio shows a very different pattern of replacement mutations compared with IgG, with a low R/S ratio in the CDRs (<1) and a greater ratio in the framework regions 2 and 3 (<4) (Fig. 5b). A similar pattern is observed in IgM sequences from immunized mice with depleted GCs (Fig. 5c). Based on the distribution of mutations and the R/S ratios, it is unlikely that IgM LLPCs are antigen selected as in IgG and our results may indicate the necessity of the GC in antigen selection.

### IgM LLPCs provide protective immunity

**Figure 6:**
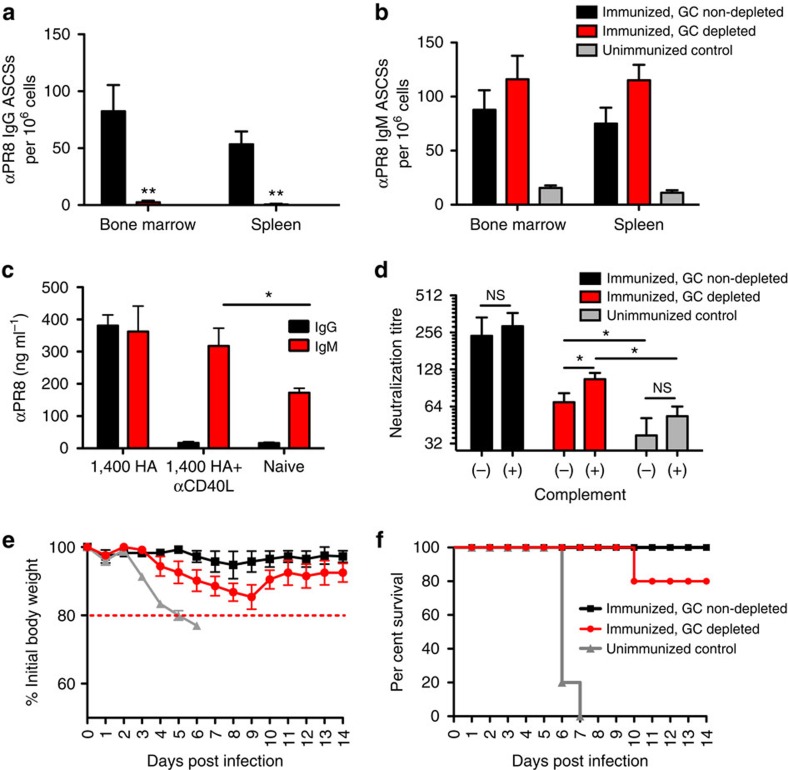
GC independent IgM LLPCs are capable of neutralizing influenza virus *in vitro* and protecting the animal against infection *in vivo.* (**a**,**b**) PR8-specific IgG (filled black square) and IgM (filled red square) ASCs, respectively, as measured by ELISPOT from cohorts 2 months post immunization with 1,400 HA PR8 (*n*=5)—treated with control Ig or αCD40L at days 6, 8 and 10 to inhibit GCs—or age-matched naive control mice (filled grey square open square *n*=3). (**c**) Antigen-specific IgG (filled black square) and IgM (filled red square) titres as measured by serum ELISA at 150 days post immunization, with or without αCD40L treatment. (**d**) Neutralization of PR8 virus by day 150 sera from PR8-immunized cohorts (*n*=5), with (filled red square) or without (filled black square) αCD40L treatment, or age-matched naive controls (filled grey square open square *n*=3), with or without complement; Student's *t*-test **P<0.005. (**e**,**f**) Mice immunized with A/PR/8/34 virus, with or without αCD40L treatment, were challenged with 10xLD_50_ of virus 1 year post immunization, with morbidity (body weight loss) and survival rate measured, respectively. The mean (±s.e.m.) of five mice in each cohort is shown.

We next sought to determine whether IgM antibodies from LLPCs could aid in host protection against a viral pathogen. In influenza infection, the antibody response has long been regarded as the key correlate of protection and it is critical to the neutralization of the virus^27^. To assess protection, we first immunized cohorts of mice with betapropiolactone (BPL)-inactivated A/PR/8/34 influenza virus. We then treated one cohort with αCD40L antibody, to deplete GCs and IgG LLPCs, such that these cohorts would produce only IgM LLPCs. We verified that IgG LLPCs were ablated in αCD40L-treated cohorts (Fig. 6a), whereas PR8-specific IgM LLPCs persisted at greater numbers than in naive controls (Fig. 6b). Sera taken at day 150 post immunization confirmed the absence of IgG antibodies following αCD40L treatment (Fig. 6c). This sera was then assessed for neutralization of PR8 virus and compared with sera from mice with intact GCs, which had both IgG and IgM LLPCs, and naive controls (Fig. 6d). Sera from αCD40L-treated mice, which was predominantly IgM, effectively neutralized PR8 virus—in particular with addition of complement. IgM from naive mice was significantly less effective, even with the addition of complement.

We further show that induced IgM from LLPCs is more effective than natural IgM in naive mice in protecting against lethal viral challenge *in vivo*. At 1 year post immunization, cohorts of immunized mice—with and without GC inhibition—and age-matched unimmunized controls were challenged intranasally with live, mouse-adapted 10xLD_50_ PR8 virus. Mice were weighed daily and those animals losing >20% of their initial body weights were killed (Fig. 6e). Following lethal virus challenge, all of the naive control mice lost >20% of their initial body weight and had to be killed by day 7. Immunized mice with intact GCs and IgG LLPCs exhibited 100% survival with minimal morbidity. The αCD40L-treated cohort, with IgM but not IgG LLPCs, showed an 80% survival rate—thus demonstrating effective protection (Fig. 6f). All surviving mice, with or without GC blockade, began to recover body mass by day 10 (Fig. 6e). Taken together, these data demonstrate that mice lacking IgG LLPCs are still effectively protected against lethal influenza virus challenge up to 1 year post immunization, and that IgM LLPCs enhance host protection in their absence, whereas natural IgM alone is not sufficient to protect naive mice *in vivo*. However, memory T cells and GC-independent memory B cells may also have played a role in viral clearance^28^.

**Figure 7:**
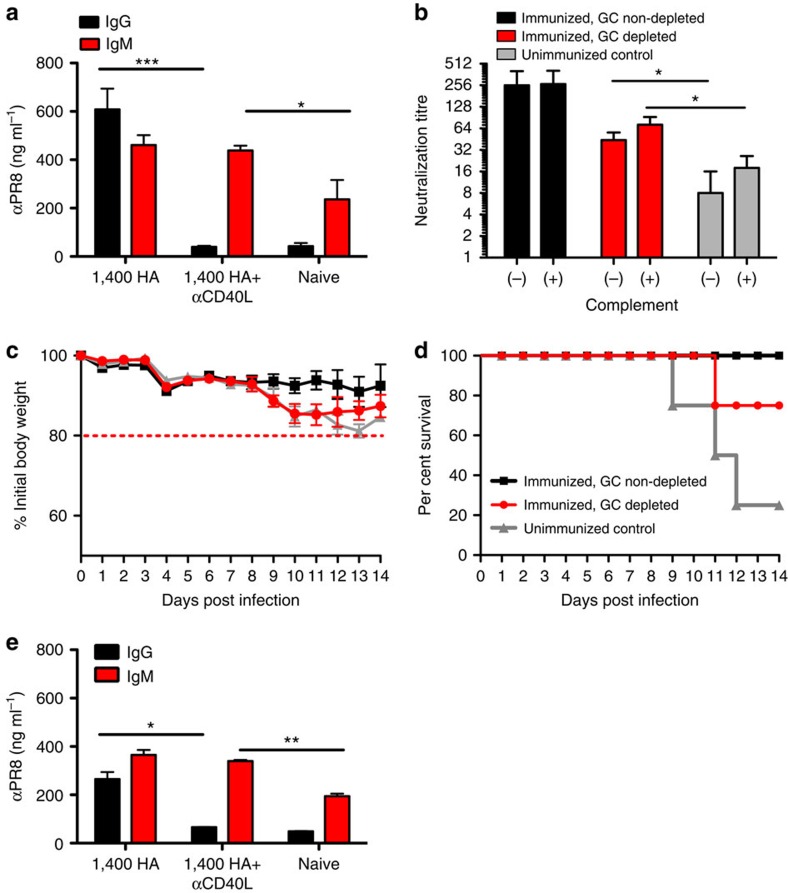
IgM LLPCs are capable of protecting the animal against infection *in vivo* in the absence of IgG PCs, memory B cells, and T cell help. (**a**) PR8-specific IgG (filled black square) and IgM (filled red square) titres, respectively, as measured via serum ELISA from cohorts at 1 month post infection with 0.1xLD_50_ PR8 virus—treated with control Ig or αCD40L at days 6, 8 and 10 to inhibit GCs—or age-matched naive control mice (filled grey square open square *n*=4). (**b**) Neutralization of PR8 virus by 1 months sera from PR8-immunized cohorts, with (filled red square) or without (filled black square) αCD40L treatment, or age-matched naive controls (filled grey square open square *n*=4), with or without complement. Mice immunized with A/PR/8/34 virus, with or without αCD40L treatment, were then treated with anti-CD4/CD8 at 1 and 3 days before infection and challenged with 2xLD_50_ of virus at 2 months post immunization. (**c**,**d**), Morbidity (body weight loss) and survival rate measured, respectively, post-PR8 challenge. The mean (±s.e.m.) of four mice in each cohort is shown. (**e**) PR8-specific IgG (filled black square) and IgM (filled red square) titres, respectively, as measured via ELISA 14 days post-PR8 challenge. *P<0.05, **P<0.005, and ***P<0.001, Student's *t*-test.

To rule out the contribution of memory B and T cells, we repeated this experiment and additionally depleted both CD4 and CD8 T cells before challenge, thereby preventing reactivation of memory B cells^29^. Thus, we could assess the role of antibody alone in protection. Briefly, mice were first infected intranasally with 0.1xLD_50_ of live mouse-adapted PR8 virus, with one cohort depleted of GCs and IgG LLPCs via αCD40L. Sera from these mice were collected at 1 month and assessed for the presence of αPR8 IgG and IgM, as well as neutralization (Fig. 7a,b). Similar to our previous experiment (Fig. 6c,d), we do not see significant IgG in either GC-depleted mice or in age-matched unimmunized controls. Higher titres of IgM in immunized/GC-depleted sera equated with significantly higher neutralization titres when compared with unimmunized mice, in particular when complement was added. We then depleted T cells from all cohorts and challenged mice with virus. At 1 and 3 days before viral challenge, mice were treated with 500 μg anti-CD4 and -CD8, effectively depleting the T cells, as well as preventing memory B-cell reactivation (Supplementary Fig. 3). Mice were challenged with 2xLD_50_ of live mouse-adapted PR8 virus—the lower dosage ensuring that immunized cohorts would survive long enough for post-challenge assessment. Once again all immunized/non-GC-depleted mice that bear virus-specific IgG and IgM antibodies survived the challenge, whereas all of the unimmunized controls succumbed to infection and 75% of the mice had to be killed (Fig. 7d). Conversely, 75% of the GC-depleted cohort that bears only virus-specific IgM survived (Fig. 7c,d). Following PR8 challenge, titres of PR8-specific IgG were measured by serum ELISA, to determine whether reactivation of memory B cells had occurred. No significant IgG titres were observed in the post challenge sera of αCD40L-treated mice (Fig. 7e), indicating that protection was due to IgM from LLPCs and not from reactivation of memory B or T cells.

## Discussion

In conclusion, our studies have characterized a distinct subset of long-lived, induced IgM LLPCs that persist within the spleen. Unlike bone marrow-resident IgG LLPCs, these cells can develop in the absence of GCs. With the aid of high-throughput sequence analysis we have shown that these cells are somatically mutated, although the lower number of mutations in the absence of GC may indicate it still plays some role in maturation. Mutations in IgM LLPCs were found in AID-induced ‘hotspots', but did not show any evidence of antigen selection for enriched CDR mutations. Further, we demonstrate that these IgM LLPCs are functionally relevant and, even in the absence of IgG LLPCs and memory B and T cells, protect *in vivo* against lethal influenza challenge.

The longevity and distinct localization of IgM LLPCs highlight the spleen as a unique niche for predominantly IgM LLPCs. Previous studies by Ahmed and colleagues^1^ have noted the presence of antigen-specific IgG LLPCs in the spleen—originally observed in response to LCMV infection in mice—but they did not characterize IgM LLPCs. Here we demonstrate that IgM PCs in the spleen also have long half-lives. MacLennan and colleagues^30^ have characterized the longevity and SHM profile of IgG^+^ and IgG^*−*^ PCs in response to NPCGG immunization in the spleen, but only up to day 21 post immunization and not long term. These IgG^*−*^ PCs may be similar to the long-lived IgM populations we have observed at much later time points post immunization in our study. In addition, our LCMV infection data demonstrates that virus-specific IgM LLPCs are the predominant population in the spleen for up to 2 years post infection, significantly higher than the splenic IgG LLPCs (Fig. 1h). This was also seen in immunization with NP_22_CGG and influenza. Interestingly, this preferential localization was recapitulated following adoptive transfer of LLPCs into B-cell-deficient μMTs (Fig. 2f,g), wherein IgM PCs were retained primarily in the spleen.

These induced IgM LLPCs that we have described are distinct from natural IgM cells. Although natural IgM populations remain steady over time, as we observe in our naive controls, we see a sharp initial increase and then a gradual decay of induced IgM LLPCs in the spleen following stimulation, and in the bone marrow to a lesser degree (Fig. 1a–f). There is also evidence of the expansion of NP-specific VH186.2 clones following immunization (Fig. 3e,f, h,i), whereas natural IgM is not thought to expand in response to antigen stimulation^31^. The fact that no NP-specific VH186.2 clones were found in the CD138^+^B220^*−*^ PC compartment of unimmunized mice further emphasizes that IgM LLPCs are antigen induced. In addition, IgM LLPCs are somatically mutated (Fig. 3c), further distinguishing them from either short-lived PCs or natural IgM cells. Taken together, these data demonstrate that the induced IgM LLPC compartment of the spleen is distinct from not only bone marrow-resident IgG LLPCs but also natural IgM cells^12^,^32^.

In addition, we have shown that IgM LLPCs were able to develop in the absence of GC formation, unlike IgG LLPCs (Figs 3a,b and 6a,b, and Supplementary Fig. 2). This parallels the discovery of GC-independent IgM memory B cells, which develop outside of or in the absence of GCs^28^. Jenkins and colleagues^33^ demonstrated that IgM memory B cells are still able to develop in *Bcl6*^*−/−*^ mice, which lack GCs. They also observed in wild-type mice that IgM memory cells were somatically mutated, although less frequently than IgG cells. The occurrence of SHM outside of GCs has previously been observed in B cells in autoimmune hosts. Schlomchik and colleagues^34^ demonstrated in autoimmune MRL.Fas(lpr) mice that autoreactive B cells, microdissected from the border of the T-cell zone and red pulp, had undergone SHM outside of GCs, although GCs were present in this model. They also recently demonstrated extrafollicular SHM in plasmablasts in response to *Salmonella* infection, without significant GC formation^35^. In human patients suffering from hyper-IgM disorder due to a defect in CD40L, which inhibits GC function, low levels of SHM are observed in memory B cells^36^. Here we directly compare the frequency and position of GC-independent mutations from hundreds of sequences. We have additionally controlled for any potential error introduced by the sequencing method by running unmutated B220^+^CD138^*−*^CD19^+^CD25^+^IgM^*−*^CD43^*−*^ pre-B cells as a control alongside thousands of sample PCs. In comparison with the pre-B sequences from the same animals, we found that IgM LLPCs exhibit low but significant levels of SHM in the Ig variable loci, even in the absence of GC formation (Fig. 3c). However, we do find a significantly lower frequency of IgM mutations in mice with impaired GCs. With or without the GC, these mutations occurred primarily in AID-induced SHM hotspots and consisted frequently of transitions, typical of AID induction (Fig. 4a–d). We do not observe these mutations in AID^*−*/*−*^ cohorts, confirming that they are purely AID dependent (Fig. 4e). Further, the fact that these mutations do not show evidence of antigen selection indicates the necessity of the GC in this process (Fig. 5a–d). It is possible that these IgM plasma cells may still undergo some alternative form of antigen selection, but it is unlike the mechanism we see in IgG LLPCs in which the CDRs are enriched for replacement mutations.

Functionally, IgM antibodies can confer the host with protection against viral and bacterial infections^37^,^38^,^39^,^40^. Monoclonal IgM antibodies isolated from human patients have been shown to protect mice against a lethal challenge of H5N1 and H1N1 influenza viruses^39^. This monoclonal IgM also neutralized a diverse range of influenza subtypes^39^. The fact that IgM PCs develop in response to T-independent antigens^41^, or in the absence of the GC (Figs 3a,b and 6a,b), may imply that this pathway continues to serve as an auxiliary pathway to protect the host even in the absence of T-cell help. In this study, we demonstrate the role of IgM LLPCs in protecting against influenza viral infection in the absence of IgG LLPCs (Fig. 6). Cohorts of mice with depleted IgG responses can effectively neutralize virus and these IgM LLPC-only animals can also survive a lethal viral challenge (Fig. 6), even in the absence of T-cell help and memory B-cell activation (Fig. 7).

Another potential role for a IgM LLPCs is suggested by recent evidence showing that IgM antibodies may be necessary to generate an optimal IgG response^42^. In mice deficient for secretory IgM (sIgM^*−*/*−*^) but still able to express surface IgM, increased viremia and an impaired IgG response was observed following infection with West Nile Virus^42^. IgM titres were further able to accurately predict survival and wild-type immune sera were sufficient to rescue infected sIgM^*−*/*−*^ cohorts. It has also been shown in several studies that IgM, when coupled with antigen, may also help to sustain long-term IgG titres following vaccination, even with suboptimal doses^43^. This could be due to the ability of antigen-specific IgM to function as an effective adjuvant, as demonstrated in malarial vaccines. In addition, IgM antibodies may be important in overcoming maternal antibody's impairment of the infant immune response, as has been demonstrated with the IgM-adjuvanted malarial vaccine when administered to infant mice with immune mothers^44^. It is hypothesized that IgM's role in opsonization and complement recruitment, or its ability to stimulate innate cells, may contribute to this effect, but this remains an area of ongoing study^43^. Taken together, these studies imply that IgM may serve a non-redundant role in humoral memory, providing a broadly neutralizing response, as well as augmenting host IgG and overcoming impairment by maternal antibodies. Understanding the development of IgM LLPCs is critical to developing better vaccines and therapeutics^45^.

Evolutionarily, the emergence of IgM occurred long before that of IgG in mammals, appearing as far back as cartilaginous fish and occurring in all vertebrate species^46^. AID and its homologues similarly appeared at this early timepoint, coevolving with target immunoglobulins as a means of repertoire diversification^47^. There is evidence for SHM in the IgM heavy chain locus in sharks^48^ and in *Xenopus* (frogs)^49^. These mutations are typically at low levels and are frequently found in mutation hotspots (targeting G:C pairs), but are less frequent in the CDR regions than would be expected for antigen selection. It has been suggested that the absence of GCs in these animals prevents antigen selection^49^. We observe a similar pattern of low-level mutations in the AID-induced hotspots of murine PC IgM occurring in the absence of GCs, with no evidence of antigen selection. It is possible that these mutated IgM PCs exist as a remnant of this pre-GC pathway, which protects cartilaginous fish and cold-blooded vertebrates. Further study of this pathway will expand our knowledge of how the function of adaptive humoral responses has evolved. Altogether, these data suggest a unique role in humoral memory for IgM LLPCs within the splenic niche and adds further complexity to our knowledge of PC development.

## Methods

### Mice

C57BL/6 mice, BALB/c, B6.129S2-*Ighmtm1CGGn*/J (μMT) and B6.129P2-*Aicda*^*tm1(cre)Mnz*/J^ (AID^*−*/*−*^) mice were purchased from The Jackson Laboratory. All mice were maintained in a specific pathogen-free facility in accordance with the institutional guidelines of The Animal Care and Use Committee at Emory University. Mice were immunized or infected at 4–8 weeks of age and all controls were age- and sex-matched. Sample size was determined by the ‘resource equation' method, taking into account possible attrition during infection assays^50^. Animals were randomly assigned to treatment groups but the investigators were not blinded to the treatments or genotypes.

### Immunizations and infections

Cohorts of female C57BL/6 or AID^*−*/*−*^ mice were immunized intraperitoneally with 50 μg NP_22_CGG (Biosearch Technologies) with 50 μl alum in PBS for a total volume of 200 μl. Other cohorts were infected with 2 × 10^5^ p.f.u. of the Armstrong strain of LCMV. Balb/c mice were immunized intramuscularly with 1,400 hemagglutinin (HA), infected intranasally with 0.01xLD_50_, or rechallenged with 10xLD_50_ of A/Puerto Rico/8/1934 (PR8) virus. Sera, bone marrow and spleens were collected at given time points post immunization.

### Plasma cell ELISPOTs

Ninety-six-well plates were coated with 20 μg ml^−1^ NP_22_CGG or OVA, 512 HA units per mL PR8 virus, or 10 μg ml^−1^ LCMV virus and blocked with complete RPMI (10% fetal bovine serum, 1% penicillin/streptomycin, 1% HEPES and 50 μM 2-mercaptoethanol). Bone marrow and splenic cells isolated from immunized mice were serially diluted in complete RPMI and incubated 16 h at 37 °C. The plates were then treated with either anti-IgG- or -IgM-biotin (Southern Biotechnology) followed by incubation with streptavidin-alkaline phosphatase (Sigma). Plates were then developed with 5-bromo-4-chloro-3-indolylphosphate (Sigma) until spots developed and spots counted with CTL Immunospot software.

### Adoptive transfer of LLPCs

Bone marrow or splenocytes were enriched for B cells via EasySep B-cell enrichment kit (Stemcell Technologies) and CD138^+^B220^*−*^ PCs were FACS sorted from C57BL/6 mice 2 months post immunization. Marrow or splenic PCs (10^5^) were then transferred via tail-vein injection into cohorts of sex-matched μMT mice or were measured for NP_22_CGG specificity via ELISPOT. Serum antibody titres were measured by ELISA at days 7, 14, 21, 30 and 60. Plates were coated with 20 μg ml^−1^ NP_22_CGG (or 2 μg ml^−1^ anti-IgG or -IgM for standard wells) and blocked with 3% BSA. Sera from recipient μMT mice was serially diluted in PBS and incubated for 2 h. Plates were then treated with either anti-IgG–horseradish peroxidase or -IgM-biotin, followed by incubation with streptavidin–horseradish peroxidase for IgM plates (Southern Biotechnology). TMB substrate (Pierce) was added for 30 s and then the reaction stopped with 0.16 M sulfuric acid. Plates were read at 450 nm. PC numbers in the bone marrow and spleen were assayed by ELISPOT at days 7, 14, 30 and at 2 months post adoptive transfer as previously described, or at 2 months for bone marrow transfer cohorts. Half-lives were determined by linear regression of transferred cells, comparing the slopes for statistically significant differences (Graphpad Prism Version 5.0c).

### High-throughput heavy chain sequencing

Total PCs (CD138^+^B220^*−*^) and bone marrow pre-B cells (B220^+^CD138^*−*^CD19^+^CD25^+^IgM^*−*^CD43^*−*^) were isolated via cell sorting from the marrow and spleens of C57/BL/6 or AID^*−*/*−*^ mice 1 or 3 months post immunization with NP_22_CGG (Supplementary Table 1), as well as from unimmunized controls. RNA from these cells was isolated with the RNeasy kit (Qiagen) and transcribed into complementary DNA with ThermoScript RT–PCR System for First-Strand cDNA Synthesis (Invitrogen). cDNA was then amplified using the consensus heavy chain primers and PCR protocol as described by Tiller *et al*.^51^, and the *IgHV186.2*-specific primer as described by Jacob *et al*.^16^, with the second PCR amplification primers adapted and barcoded for 454 pyrosequencing. Barcoded products were amplified on sequencing beads using the GS Junior Titanium Lib-A emPCR Kit (Roche) and sequenced on the GS Junior 454 sequencer using the matching kit (Roche). Sequences were then analysed for heavy chain homology and mutations using The International Immunogenetics Information System HighV-QUEST (IMGT.org)^18^. Sequences with >90% identity with *IgHV186.2* are numbered in Supplementary Table 1. Partial or non-productive sequences were excluded from analysis.

### GC disruption

Cohorts of mice were treated with either 300 μg αCD40L (clone MR-1) antibody or control IgG at days 6, 8 and 10 post NP_22_CGG immunization. Additional cohorts were treated with 25 μg CVF or control Ig 6 h before immunization and again at 4 days post immunization. At 45 days post immunization, bone marrow cells and splenocytes were assayed by ELISPOT.

### IgTree analysis

Ig lineage trees were made using IgTree software^20^, information for which can be found at http://immsilico2.lnx.biu.ac.il/Software.html. Clones were identified by their unique CDR3 sequences and measured against the germline sequence to generate trees for the variable regions only. Boundaries were defined by the beginning and end of alignment with *IgHV186.2* sequence.

### SHM hotspot analysis

*IgHV186.2* sequences were partitioned into clonally related groups based on shared CDR3 junctions. To avoid double-counting mutational events, a consensus mutated sequence was generated for each clone through majority vote at each mutated position. The positions differed from IMGT-inferred germline segments in the V region up to the start CDR3 were considered mutations for substitution matrices and hotspot analysis. The 5-mer motif mutability values were generated by computing the mutation level of each motif adjusted for background frequency. The model and source code can be found at http://clip.med.yale.edu/SHM^24^.

### Virus neutralization

Cohorts of Balb/c mice were immunized intramuscularly with 1,400 HA of PR8 virus and treated with either αCD40L or control Ig as previously described. At 2 months post immunization, five mice from each cohort were killed and PR8-specific PCs were measured by ELISPOT for IgG ablation in αCD40L blockade mice. At day 150 post immunization, sera was collected from remaining ten mice and measured by ELISA (plates coated with 512 HA PR8) to assess IgG clearance from the sera of the αCD40L cohort. RDE II-treated sera from either cohort or age-matched naive controls, with or without complement added (1:1 ratio), was then assessed for neutralization of 2 × 10^3^ TCID_50_ per ml of PR8 virus when incubated with MDCK cells overnight at 37 °C. The presence of viral nucleoprotein in infected cells was determined by ELISA, as previously described, using anti-nucleoprotein biotinylated antibody (Chemicon International). The highest serum dilution where less than half of the cells were infected was determined to be the neutralization titre, where 50% specific signal=(OD_450_ virus control−OD_450_ cell control)/2+OD_450_ cell control)^38^.

### Viral challenge

At 1 year post immunization with 1,400 HA of virus, cohorts of PR8-immunized mice, with or without αCD40L blockade, as well as age-matched unimmunized controls were infected intranasally with 10xLD_50_ of PR8 virus. Similarly, at 2 months post infection with 0.1xLD_50_ of virus, cohorts were challenged intranasally with 2xLD_50_ of virus following T-cell depletion (described below). Mice were weighed each day for 14 days and those falling below 80% of their initial weight were killed.

### T-cell depletion

At 3 days and 1 day before viral challenge, all cohorts of mice were treated intraperitoneally with 500 μg each of both anti-CD4 (clone GK1.5) and anti-CD8a (YTS 169.4) (BioXcell)^52^,^53^. Blood samples from each mouse were then assessed for the presence of CD4 and CD8 T cells by flow cytometry. Cells were fluorescently labelled with anti-CD4 (clone RM4-5, ebioscience) and anti-CD8 (clone 53-6.7, BD) at a dilution of 1:200 in FACS buffer (1% fetal bovine serum in PBS). Cells were then analysed using the BD FACSCalibur and flow plots created in FlowJo 8.7.

### Statistical analysis

Statistical significance was verified by the Student's *t*-test (two-tailed), unless otherwise indicated.

### Data availability

The data described in this publication have been deposited in NCBI's Gene Expression Omnibus^54^ and are accessible through NCBI's GEO Series accession number GSE80405 (https://www.ncbi.nlm.nih.gov/geo/query/acc.cgi?acc=GSE80405).

## Additional information

**How to cite this article:** Bohannon, C. *et al*. Long-lived antigen-induced IgM plasma cells demonstrate somatic mutations and contribute to long-term protection. *Nat. Commun.* 7:11826 doi: 10.1038/ncomms11826 (2016).

## Supplementary Material

Supplementary Information Supplementary Figures 1 - 3 and Supplementary Table 1
