# Corrigendum: Long-lived antigen-induced IgM plasma cells demonstrate somatic mutations and contribute to long-term protection

**DOI:** 10.1038/ncomms12687

**Published:** 2016-08-16

**Authors:** Caitlin Bohannon, Ryan Powers, Lakshmipriyadarshini Satyabhama, Ang Cui, Christopher Tipton, Miri Michaeli, Ioanna Skountzou, Robert S. Mittler, Steven H. Kleinstein, Ramit Mehr, Frances Eun-Hyung Lee, Ignacio Sanz, Joshy Jacob

Nature Communications 7: Article number: 11826 10.1038/ncomms11826 (2016); Published: 06 07 2016; Updated: 08 16 2016

The original version of this Article contained an error in the spelling of the author Frances Eun-Hyung Lee that was incorrectly given as Francis Eun-Hyung Lee. This has now been corrected in both the PDF and HTML versions of the Article.

