# Supplementary material for: Long-lived antigen-induced IgM plasma cells demonstrate somatic mutations and contribute to long-term protection
Source: Nat Commun. 2016 Jun 7;7:11826. doi: 10.1038/ncomms11826 (PMC4899631; doi:10.1038/ncomms11826)
Supplement: Supplementary Information — Supplementary Figures 1 - 3 and Supplementary Table 1 [file ncomms11826-s1.pdf]

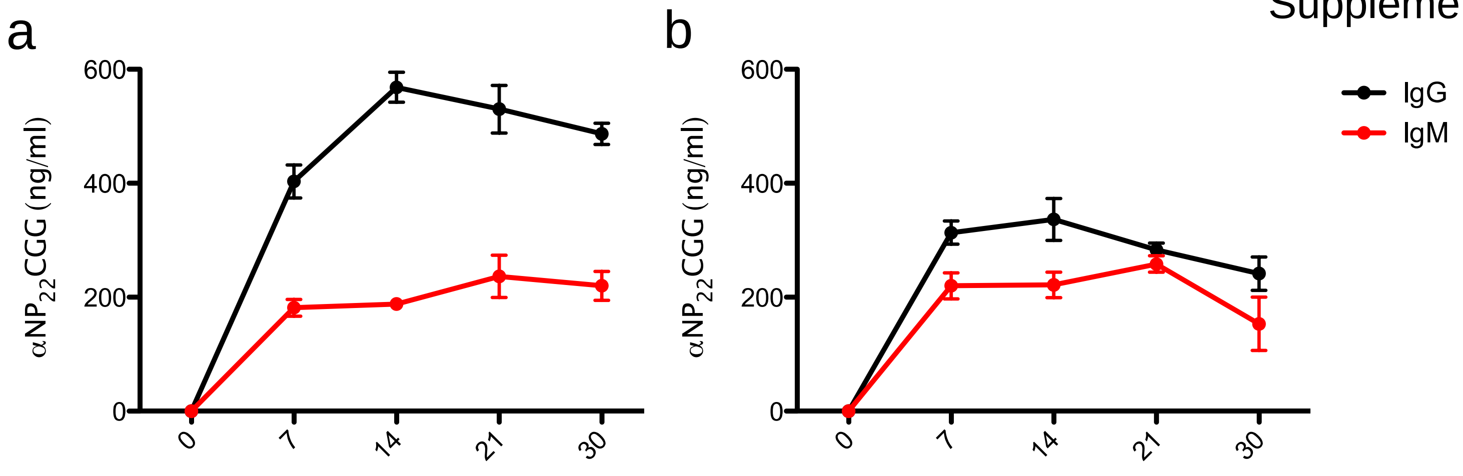

Figure 1: Antigen-specific IgM and IgG titers persist, post-adoptive transfer of plasma cells into recipient Rag-/- mice. C57/BL mice were immunized with $NP_{22}CGG$, and then 2 months later 10,000 bone marrow or splenic plasma cells were sorted and adoptively transferred into Rag-/- mice. The recipients were then bled on days 7, 14, 21, and 30 and serum levels of anti-NP antibodies were measured by ELISA. **(a-b)** Antigen-specific serum IgG (●) and IgM (●) titers found in bone marrow or splenic plasma cell recipient mice (n=3), respectively, as measured by ELISA at days 7 through 30.

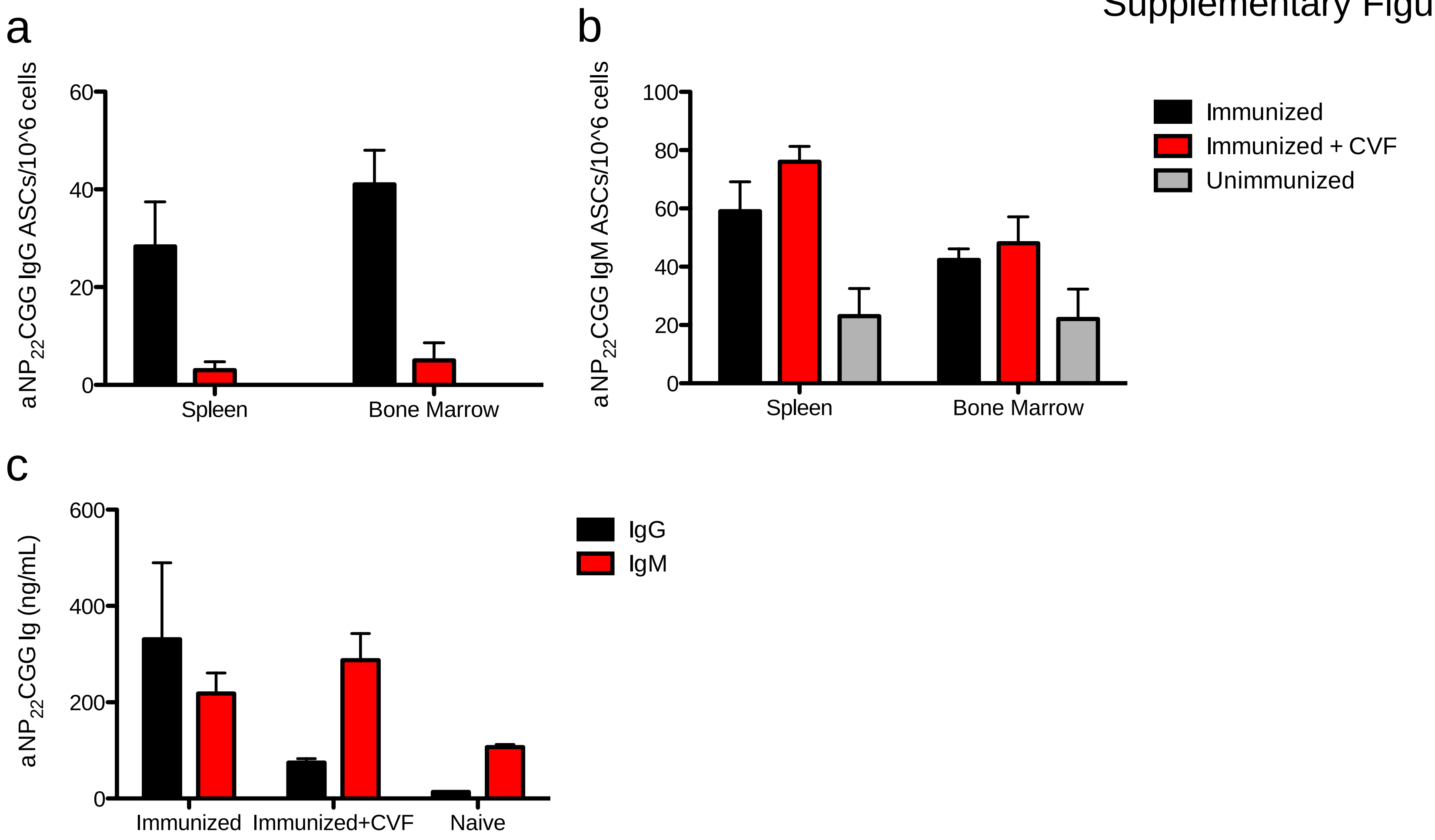

Fig 2. Long-lived IgM, but not IgG, plasma cells are generated when germinal center formation is blocked by Cobra Venom Factor (CVF) treatment. Cohorts of mice were treated with CVF at 6 hours prior to and 7 days following $NP_{22}CGG$ immunization, to deplete complement and prevent germinal center formation. Untreated, immunized mice have intact germinal centers. **(a-b)** $NP_{22}CGG$-specific IgG and IgM ASCs, respectively, in immunized mouse cohorts (n=5), with (■) or without (■) CVF treatment, or in age-matched naïve controls (■, n=3), as measured via ELISPOT at day 45 post-immunization. **(c)** $NP_{22}CGG$-specific IgG (■) and IgM (■) antibodies in the sera as measured by ELISA. The mean (±SEM) is shown with * indicating p<0.05 and ** indicating p<0.005.

Supplementary Figure 3 (Jacob)

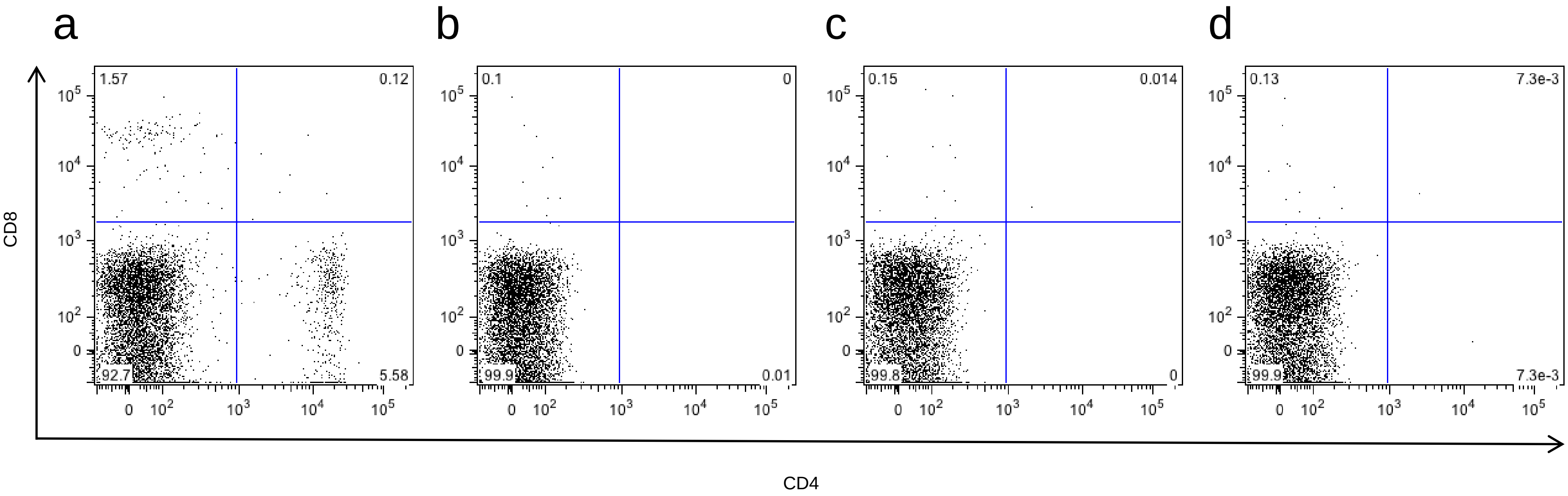

Fig. 3. Treatment with anti CD4 and CD8 antibodies leads to efficient depletion of CD4 and CD8 T cell. Representative flow plots of PBMC in control mice (n=3) and those treated with anti-CD4/CD8 Abs at 1 and 3 days prior to infection (n=4). **(a)** Untreated controls with healthy T cell populations. **(b-d)** Treated mice: PR8-immunized mice **(c)** with and **(b)** without αCD40L germinal center depletion, and **(d)** age-matched unimmunized controls.

			IgM		IgG	
			# of sequences	# of unique CDR3s	# of sequences	# of unique CDR3s
Wild type	Mouse 1	Spleen	18	8	N/A	N/A
	(1 month)	Marrow	161	14	66	6
	Mouse 2	Spleen	42	11	N/A	N/A
	(1 month)	Marrow	56	21	10	2
	Mouse 3	Spleen	32	14	N/A	N/A
	(3 months)	Marrow	15	5	9	2
	Mouse 4	Spleen	25	23	N/A	N/A
	(3 months)	Marrow	12	7	79	8
αCD40L depleted	Mouse 1	Spleen	26	21		
	(1 month)	Marrow	53	18		
	Mouse 2	Spleen	17	13		
	(1 month)	Marrow	34	19		
	Mouse 3	Spleen	27	17		
	(3 months)	Marrow	N/A	N/A		
	Mouse 4	Spleen	15	12		
	(3 months)	Marrow	2	2		
AID-/-	Mouse 1	Spleen	10	8		
	(1 month)	Marrow	3	3		
	Mouse 2	Spleen	15	10		
	(1 month)	Marrow	7	3		
	Mouse 3	Spleen	8	5		
	(1 month)	Marrow	3	1		

Table 1: Total heavy chain sequences with >90% homology to IgHV186.2. Total number of IgHV186.2 sequences and unique complementary determining region 3 (CDR3) sequences are shown from individual mice. Sequences were amplified from pooled $CD138^{+}B220^{-}$ plasma cells using a VH186.2-specific primer.
